# Adjuvant injection therapies following knee cartilage repair demonstrate heterogeneous evidence: A systematic review

**DOI:** 10.1002/jeo2.70555

**Published:** 2025-11-14

**Authors:** Max Alfredo Saráchaga Mendoza, Philipp Niemeyer, Alexander Bumberger, Peter Angele, Philip P. Roessler

**Affiliations:** ^1^ Department of Orthopaedic Surgery Hospital General de México Dr. Eduardo Liceaga Mexico City Mexico; ^2^ Hospital Ángeles del Pedregal Mexico City Mexico; ^3^ Orthopädische Chirurgie München (OCM) Munich Germany; ^4^ Albert‐Ludwigs‐University Freiburg Germany; ^5^ Department of Orthopedics and Trauma Surgery Medical University of Vienna Vienna Austria; ^6^ Sporthopaedicum Regensburg/Straubing Regensburg Germany; ^7^ University Medical Center Regensburg Regensburg Germany; ^8^ Gelenkzentrum Mittelrhein Koblenz Germany; ^9^ Department of Orthopaedics and Trauma Surgery University Hospital Bonn Bonn Germany

**Keywords:** cartilage defects, hyaluronic acid, mesenchymal stromal cells, microfractures, platelet‐rich plasma

## Abstract

**Purpose:**

The objective of this study was to systematically review the existing literature related to adjuvant injection therapies among patients with cartilage defects of the knee joint who have undergone bone marrow stimulation procedures and ascertain their potential clinical benefits.

**Methods:**

Following the Preferred Reporting Items for Systematic Reviews and Meta‐Analyses (PRISMA) guidelines, a search was conducted on PubMed and the Cochrane Library to identify articles documenting the utilization of adjuvant injection therapies after surgery for cartilage defects, with a minimum short‐term follow‐up. Animal studies, uncontrolled trials, studies lacking primary surgical intervention or those including patients with osteoarthritis were excluded.

**Results:**

Twelve articles focusing on knee cartilage defects were analysed (531 patients). Platelet‐rich plasma was used in eight studies, mesenchymal stromal cells in four and hyaluronic acid in one. Injection frequency and timing varied, with the most common timing being during surgery. Primary outcome measures included subjective International Knee Documentation Committee (IKDC) and Visual Analogue Scale (VAS) scores. Subjective IKDC showed significant difference in five out of nine studies and VAS in three out of eight studies, favouring injection therapy groups.

**Conclusion:**

The present review demonstrates a large heterogeneity among included studies regarding surgical interventions, injection strategies and timing as well as outcome measures. While several studies suggest a potential benefit of adjuvant therapies, findings remain inconsistent. Due to the limited quality and comparability of the evidence, no definitive recommendations can be made at this time, highlighting the need for standardized protocols and high‐quality randomized controlled trials.

**Level of Evidence:**

Level IV.

## Graphical abstract

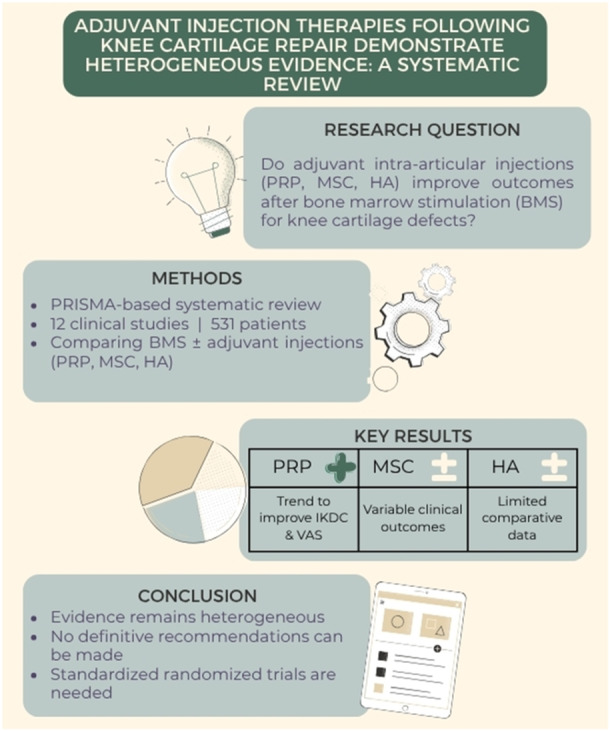

**Abbreviations**
ACI: autologous chondrocyte implantation
ADMSC: adipose‐derived mesenchymal stromal cells
BMAC: bone marrow aspirate concentrate
BMDMSC: bone marrow‐derived mesenchymal stromal cells
BMS: bone marrow stimulation
FU: follow‐up
HU: hyaluronic acid
ICRS: International Cartilage Repair Society
IKDC: International Knee Documentation Committee
KOOS: Knee Injury and Osteoarthritis Outcome Score
MF: microfractures
MRI: magnetic resonance imaging
MSC: mesenchymal stromal cells
OA: osteoarthritis
OAT: osteochondral autograft transfer
OCT: osteochondral allograft transplantation
PBSC: peripheral blood stromal cells
PRF: platelet‐rich fibrin
PRISMA: Preferred Reporting Items for Systematic Reviews and Meta‐Analyses
PROM: patient‐reported outcome measures
PRP: platelet‐rich plasma
RCT: randomized controlled trial
VAS: Visual Analogue Scale
WOMAC: Western Ontario and McMaster Universities osteoarthritis index

## INTRODUCTION

Surgical treatment options for focal cartilage defects mainly depend on defect size and localization [14]. The most common procedures are bone marrow stimulation (BMS) techniques like microfractures (MF), osteochondral autograft transfer (OAT), osteochondral allograft transplantation (OCT) as well as autologous chondrocyte implantation (ACI) [9]. With the appropriate indication and patient selection, all of these methods have reported good to excellent clinical results [1, 10, 26, 48]. An alternative approach involves using scaffolds to stabilize the clot formed and optimizing the conditions for tissue repair. Some augmentation options are collagen‐based scaffolds [52, 53], hyaluronic acid (HA)‐matrices [50] or hydrogels [42], which have demonstrated improvements in the outcomes of MF alone. There is no standardized aftercare following these procedures, and rehabilitation protocols as well as adjuvant therapy strategies remain at the discretion of the surgeon [5, 9, 14].

Orthobiologics such as platelet‐rich plasma (PRP), bone marrow aspirate concentrate (BMAC) and mesenchymal stromal cells (MSC) are increasingly used for conditions such as osteoarthritis (OA), cartilage, tendon and ligament injuries. They have shown promising results in improving function and reducing pain, particularly in cartilage repair, regardless of patient age or lesion size during mid‐term follow‐up (FU) [36].

PRP, with its high platelet content, releases growth factors at injury sites promoting regeneration, reducing inflammation and enhancing tissue repair, while its preparation is straightforward and cost‐effective, though the optimal protocol remains uncertain [17, 49]. MSCs have shown encouraging results for therapeutic use, due to their regenerative abilities and modulation of immune response, with studies suggesting positive outcomes for OA, indicating the potential of MSC‐based therapies [19, 57]. HA, naturally present in joints, offers therapeutic benefits like shock absorption, lubrication, anti‐inflammatory effects and chondroprotection for treating cartilage lesions or OA [3, 8, 47].

This review aimed to identify possible benefits of adjuvant injection therapies following surgical treatment of cartilage defects. Orthobiologic approaches like PRP, HA as well as MSC were investigated for their effects in addition to predefined standard surgical procedures. The hypothesis was that adjuvant injections, particularly with PRP, would show superior clinical outcomes when compared with controls, although the heterogeneity of protocols would make it difficult to give definitive recommendations.

## MATERIALS AND METHODS

### Search strategy

A systematic review was conducted according to PRISMA (Preferred Reporting Items for Systematic Reviews and Meta‐Analyses) guidelines. All available studies evaluating the effect of adjuvant injection therapy in the context of surgical treatment of cartilage defects were screened for eligibility. The last queries were performed in PubMed and the Cochrane Library on 15 May 2025 by two independent authors.

The following search string was used: (microfracture OR ‘bone marrow stimulation’ OR chondroplasty OR ‘osteochondral transplantation’ OR mosaicplasty OR ‘chondrocyte transplantation’) AND (PRP OR ‘platelet‐rich plasma’ OR ‘plasma rich in growth factors’ OR ‘platelet‐derived’ OR ‘platelet concentrate’ OR PRF OR ‘platelet rich fibrin’ OR ‘platelet lysate’ OR ‘thrombocyte concentrate’ OR viscosupplements OR ‘hyaluronic acid’ OR hyaluronan OR hyaluronate OR ‘mesenchymal stem cells’ OR ‘stromal cells’ OR ‘stromal vascular fraction’ OR ‘adipose‐derived’ OR ‘bone‐marrow‐derived’).

### Screening and inclusion criteria

All articles were screened by title and extended abstract. Inclusion criteria were defined as follows: clinical trials, randomized controlled trials (RCTs) or case control and cohort studies on the use of PRP, HA, MSC (or a combination of the latter) as an adjuvant injection therapy following surgical treatment of cartilage defects of the knee joint or at the time of surgery (e.g., BMS techniques, OAT, OCT or ACI) with a minimum of 6‐month FU. Animal studies and controlled laboratory studies were excluded, as well as reviews. Moreover, studies without primary surgical intervention as well as studies including patients with OA (Kellgren–Lawrence Grade > 2) were excluded. Only studies published in English were included. After screening, identified full texts were sought for retrieval.

### Methodological assessment

All retrieved full texts were assessed for eligibility and articles excluded if they did not meet the inclusion criteria. The Oxford Centre for Evidence‐Based Medicine guidelines were employed to ascertain the level of evidence for each publication. To assess the quality of each publication, the modified Coleman methodology score was used, consisting of ten criteria and yielding a score ranging from 0 to 100 (Table 1) [33].

**Table 1:** Modified Coleman score.

Part A: Only one score to be given for each of the seven sections	
Study size: number of patients	
<30	0
30–50	4
51–100	7
>100	10
Mean follow‐up, months	
>12	0
12–36	4
37–60	7
>61	10
Surgical approach	
Different approaches used and outcome not reported separately	0
Different approaches used and outcome reported separately	7
Single approach used	10
Type of study	
Retrospective cohort study	0
Prospective cohort study	10
Randomized controlled trial	15
Description of diagnosis	
Described without percentage specified	0
Described with percentage specified	5
Description of surgical technique	
Inadequate (not stated, unclear)	0
Fair (technique only stated)	5
Adequate (technique stated, details of surgical procedure given)	10
Description of postoperative rehabilitation	
Described	5
Not described	0

Part B: Scores may be given for each	
Outcome criteria	
Outcome measures clearly defined	2
Timing of outcome assessment clearly stated	2
Use of outcome criteria that has reported reliability	3
General health measure included	3
Procedure of assessing outcomes	
Participants recruited	5
Investigator independent of surgeon	4
Written assessment	3
Completion of assessment by patients	3
Description of subject selection process	
Selection criteria reported and unbiased	5
Recruitment rate reported	
>90%	5
<90%	0

### Data extraction

Subsequently, the following data from each article were extracted: first author, year of publication, journal of publication, type of cartilage treatment, type of injection therapy, number of injections, number of patients followed, FU period, study design and the outcome parameters of each study.

## RESULTS

### Study characteristics

**Figure 1:**
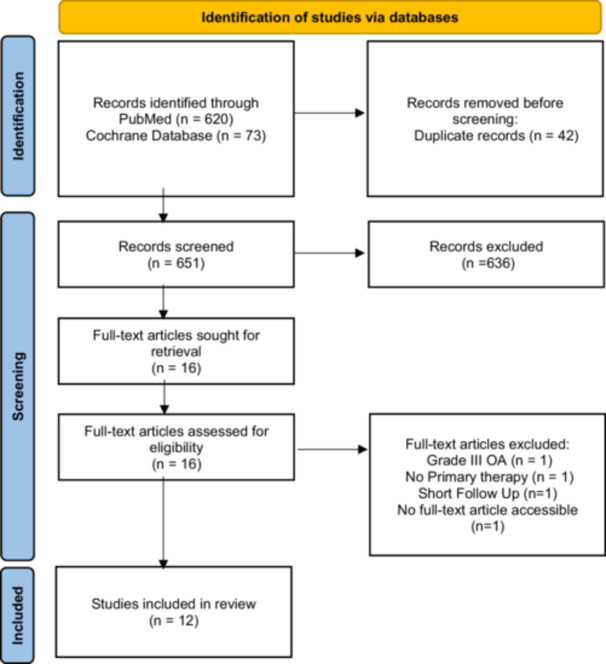
Flowchart for literature identification, following the PRISMA guidelines. PRISMA, Preferred Reporting Items for Systematic Reviews and Meta‐Analyses; OA, osteoarthritis.

A total of 12 studies were included in the final analysis (Figure 1). Among these, MF was utilized in 11 studies and chondroplasty was utilized in one study. The FU periods ranged from 48 weeks to 60 months, with the most common duration being 12 months. The total number of patients included across these studies was 531 (10–120). The most frequently used injection therapy was PRP (*n* = 8), followed by adipose‐derived mesenchymal stromal cells (ADMSC) (*n* = 2), bone marrow‐derived mesenchymal stromal cells (BMDMSC) (*n* = 1), peripheral blood stromal cells (PBSC) (*n* = 1), platelet‐rich fibrin (PRF) (*n* = 1) and HA (*n* = 1). From a methodological point of view, seven studies with Level of Evidence II were identified, two studies with Level of Evidence III and three studies with Level of Evidence IV. The mean Coleman methodology score is 59.16 (42–73) (Table 2).

**Table 2:** Studies related to knee cartilage injuries; follow‐up in months, unless specified otherwise.

Knee	Year	Journal	Type of cartilage treatment	Cartilage lesion	Type of injection therapy	Number of injections	Timing of first injection	Number of patients	Age	Follow‐up period (months)	Study design	mCMS
Saw et al. [44]	2021	Arthroscopy	MF	ICRS III/IV ≥3 cm^2^	PBSC + HA HA + physiotherapy^a^	>1 (14)	Day 7	Total: 69 Intervention: 36 Control: 33^a^	44.8 ± 8.83 [23–55] 44.6 ± 7.08 [25–55]^a^	24	RCT	61
Manunta and Manconi [35]	2013	Joints	MF	Outerbridge Grade 2–3	PRP versus control	>1 (3)	Day 7	Total: 20 PRP: 10 Control: 10	30–55	12	RCT	61
Gu et al. [21]	2023	Am J Transl Res	MF	Outerbridge 1–4	PRP versus control	>1 (3)	Day 3	Total: 120 PRP: 65 Control: 55	18–65	12	Retrospective case control	46
Lee et al. [31]	2013	Eur J Orthop Surg Traumatol	MF	K&L I/II Outerbridge III/IV <4 cm^2^	PRP versus control	1	Intraoperative	Total: 49 PRP: 24 Control: 25	40–50	24 PRP: 30.8 (24–36) Control: 32.2 (26–36)	RCT	71
Koh et al. [28]	2016	Arthroscopy	MF	ICRS III/IV ≥3 cm^2^ K&L I/II	ADMSC versus control	1	Intraoperative	Total: 80 Study: 40 Control: 40	18–50	27.4	RCT	73
Mancò et al. [34]	2016	Joints	MF	Outerbridge III/IV K&L I/II	PRP versus control	1	Intraoperative	Total: 27 Study: 14 Control: 13	52.4	24	Prospective cohort study	59
Papalia et al. [41]	2016	Journal of Biological Regulators & Homoeostatic Agents	MF	Outerbridge II/III	PRF versus PRP versus control	1	PRF: intraoperative PRP: postoperative^b^	Total: 48 PRF: 15 PRP: 16 Control: 17	53 ± 9.8 52 ± 11 53 ± 9.8	60	Retrospective case control	42
Hashimoto et al. [24]	2019	Regenerative Therapy	MF	Outerbridge III/IV K&L I/II ≥2 cm^2^	BMDMSC versus control	1	Intraoperative	Total: 11 Study: 7 Control: 4	44.1	48 weeks	RCT	60
Danieli et al. [13]	2020	International Orthopaedics	Chondroplasty	ICRS III <2 cm^2^ K&L I	PRP versus control	1	Intraoperative	Total: 64 Study: 33 Control: 31	35.65 ± 8.25	24	RCT	70
Wang and Gao [55]	2022	International Orthopedics	MF	≤4 cm^2^ <2 cm Ø	PRP	1	Intraoperative	28	31.09 [16–42]	12	Case series	56
Venosa et al. [51]	2022	Advances in Orthopedics	MF	Outerbridge IV <2 cm^2^	PRP versus PRP + ADMSC	1	Intraoperative	Total: 38 PRP: 19 PRP + ADMSC: 19	56.3 [45–73]	12	RCT	62
Brusalis et al. [12]	2020	The Knee	Augmented MF	<2.0 cm^2^	Augmentation with BioCartilage and PRP	1	Intraoperative	10	39.7 [19–66]	24	Case series	49

Abbreviations: ADMSC, adipose‐derived mesenchymal stromal cells; BMDMSC, bone marrow‐derived mesenchymal stromal cells; HA, hyaluronic acid; ICRS, International Cartilage Repair Society; mCMS, modified coleman score; MF, microfracture; PBSC, peripheral blood stromal cells; PRF, platelet‐rich fibrin; PRP, platelet‐rich plasma; RCT, randomized controlled trial.

^a^ Control group had no surgery (patients not considered for final analysis).

^b^ Timing of injection therapy is not specified.

### Clinical outcomes

Patient‐reported outcome measures (PROM) were the most frequently defined primary endpoint. The most used metrics were the subjective International Knee Documentation Committee (IKDC) score, Visual Analogue Scale (VAS), Western Ontario and McMaster Universities Osteoarthritis Index (WOMAC), SF‐36/SF‐12, Knee Injury and Osteoarthritis Outcome Score (KOOS) and Lysholm score. Subjective IKDC and VAS were the most frequently reported parameters across studies. Regarding the subjective IKDC score, at 12‐month FU, only two studies reported a significant difference between groups. However, at the 24‐month FU, three studies reported significant differences (Table 3). Regarding VAS, two studies had significant difference between groups at 12‐ and 24‐month FU (Table 4). All other scales mentioned were used in only a few of the studies. A meta‐analysis could not be performed due to the heterogeneity of study designs, outcome measurements and the way results were reported in each study. In one of the three studies where the Lysholm score was used, a statistically significant difference favouring the PRP injection‐treated group was observed at 12‐month FU [21]. Tegner scale was used in one study [13], reporting significant difference in favour of PRP injection at 12‐month FU. The KOOS scale was used in six studies with varying reporting formats, complicating direct comparisons. In one study [13], significant differences at both 12‐ and 24‐month FU were reported. Other studies [24, 28, 44] focused on specific subdomains, observing significant differences in different timeframes. Quality of life (QOL) was assessed in some studies using the SF‐12 or SF‐36 questionnaires; however, none of these studies reported a significant difference in this regard.

**Table 3:** Results for the subjective IKDC scale.

Subjective IKDC	No. of injections	First injection	Therapy	*n*	Baseline	12 months	24 months	Additional FU	Comments	
Manunta et al. [35]	>1 (3)	Day 7	MF + PRP MF	10 10	31.2 ± 4.6 30.1 ± 4.6	85.8 ± 6.9^a^ 74.3 ± 5.3			*p* = 0.001 at 12 months	d
Saw et al. [44]	>1 (14)	Day 7	MF + HA + PBSC HA + physiotherapy (no surgery)^b^	36 33	43.1 (SE 2.31) 42.7 (SE 2.37)	57.0 (SE 2.99) 52.8 (SE 3.43)	65.6 (SE 3.31)^a^ 48.1 (SE 3.34)		*p* = 0.0045 at 18 months *p* < 0.001 at 24 months Control group no surgery	^d^
Lee et al. [31]	1	Intraoperative	MF + PRP MF	24 25	49.5 [32–77] 46.7 [27–71]	76.3 [60–95] 71.5 [58–91]	87.7 [70–98]^a^ 73.2 [61–93]		*p* = 0.012 at 24 months	^d^
Papalia et al. [41]	1	PRF: intraoperative PRP: postoperative^b^	MF + PRF MF + PRP MF	15 16 17	Not reported	Not reported	85.3 ± 15.6^c^ 73.9 ± 14.3^a^ 62.7 ± 9.8	78.4 ± 13.9^a^ 76.5 ± 14.9^a^ 62.8 ± 12.8 (60 months)	PRF versus PRP *p* = 0.035, PRF versus MF *p* = 0.001, PRP versus MF *p* = 0.012 at 24 months PRF versus MF *p* = 0.002, PRP versus MF *p* = 0.007 at 60 months	^d^
Danieli et al. [13]	1	Intraoperative	Chondroplasty + PRP chondroplasty	33 31	46.1 (SE 2.5) 45.2 (SE 2.6)	84.1 (SE 1.7)^a^ 67.9 (SE 1.8)	80.5 (SE 1.7)^a^ 69.5 (SE 1.8)		*p* = 0.001 at 12 and 24 months Also, ACL versus no ACL	^d^
Mancó et al. [34]	1	Intraoperative	MF + PRP MF	14 13	34.63 ± 15 37.02 ± 12	72.10 ± 5.93 62.58 ± 9.60	67.11 ± 26.74 62.13 ± 19		No difference between groups	^e^
Brusalis et al. [12]	1	Intraoperative	MF + biocartilage and PRP	10	Not reported	~72	~78		Results are reported in figures. No comparison Improvement over time *p* < 0.01	^e^
Venosa et al. [51]	1	Intraoperative	MF + PRP MF + PRP and ADMSC	19 19	58.3 ± 2.4 59.2 ± 2.7	76.9 ± 2.8 78.2 ± 2.2			No difference between groups	^e^
Hashimoto et al. [24]	1	Intraoperative	MF + BMDMSC MF	7 4	47.1 34.5	~78 ~62			No difference between groups Results reported in figures	e

Abbreviations: ADMSC, adipose‐derived mesenchymal stromal cells; BMDMSC, bone marrow‐derived mesenchymal stromal cells; FU, follow‐up; HA, hyaluronic acid; IKDC, International Knee Documentation Committee; MF, microfracture; PBSC, peripheral blood stromal cells; PRF, platelet‐rich fibrin; PRP, platelet‐rich plasma; SE, standard error.

^a^ Statistically significant difference.

^b^ Control group had no surgery (patients not considered for final analysis).

^c^ Statistically significant difference between both intervention groups and the control group.

^d^ Statistically significant difference between groups at any point of FU.

^e^ No statistically significant difference between groups at any point of FU or no control group.

**Table 4:** Results for VAS scale.

VAS (knee)	No. of injections	First injection	Therapy	*n*	Baseline	12 months	24 months	Additional FU	Comments	
Papalia et al. [41]	1	PRF: intraoperative PRP: postoperative^a^	MF + PRF MF + PRP MF	15 16 17	Not reported		1.35 ± 1.8 3.5 ± 1.7^b^ 5.2 ± 1.6^c^	3.4 ± 1.8 3.1 ± 1.51 5.5 ± 1.5^c^ (60 months)	PRF versus PRP *p* = 0.001, PRF versus MF *p* = 0.001, PRP versus MF *p* = 0.007 at 24 months PRF versus MF *p* = 0.001, PRP versus MF *p* = 0.001 at 60 months	^d^
Gu et al. [21]	>1 (3)	Day 3	MF + PRP MF	65 55	~5.8 ~5.7	~1.2 ~2.4^b^			Results reported in figures *p* < 0.05 at 3, 6 and 12 months	^d^
Lee et al. [31]	1	Intraoperative	MF + PRP MF	24 25	8.1 [7–10] 8.5 [6–10]	3.2 [1–4] 3.6 [1–4]	2.3 [0–4] 3.4 [0–4]^b^		*p* = 0.017 at 24 months	^d^
Manunta and Manconi [35]	>1 (3)	Day 7	MF + PRP MF	10 10	8.2 ± 0.6 8.1 ± 0.6	1.4 2 ± 0.7			No difference between groups	^e^
Mancó et al. [34]	1	Intraoperative	MF + PRP MF	14 13	6.43 ± 1.91 6.62 ± 1.26	1.79 ± 0.89 3.00 ± 1.53	3.36 ± 2.84 3.54 ± 2.26		No difference between groups	^e^
Wang and Gao [55]	1	Intraoperative	PRP + fibrin gels	28	6.57 ± 1.07	2.09 ± 1.35^b^			No control group Improvement over time *p* < 0.05	^e^
Venosa et al. [51]	1	Intraoperative	MF + PRP MF + PRP and ADMSC	19 19	6.09 ± 2.33 6.19 ± 1.97	3.42 ± 2.55 3.32 ± 2.43			No difference between groups	^e^
Koh et al. [28]	1	Intraoperative	MF + ADMSC MF	40 40	Only improvement reported. Decreased significantly in both groups. Study group had significantly greater decrease than control group (*p* = 0.032) (FU 24 months)					^e^

Abbreviations: ADMSC, adipose‐derived mesenchymal stromal cells; FU, follow‐up; MF, microfracture; PRF, platelet‐rich fibrin; PRP, platelet‐rich plasma; RCT, randomized controlled trial; VAS, visual analogue scale.

^a^ Timing of injection therapy not specified.

^b^ Statistically significant difference.

^c^ Statistically significant difference between both intervention groups and the control group.

^d^ Statistically significant difference between groups at any point of FU.

^e^ No statistically significant difference between groups at any point of FU or no control group.

### Number and timing of injections

In nine of the studies, participants received a single injection. Among these, in five studies PRP was utilized as injection therapy, in two studies patients received ADMSC, in one study BMDMSC, in one study PRF and PRP were compared and in one study PRP and ADMSC were compared. In all cases, the injections were administered Intraoperative. In three studies, multiple injections were administered. In one of those, patients were treated with PBSC and HA, receiving 14 injections, and in the other two PRP was utilized as injection therapy, receiving three injections. In two studies, the first injection was administered on Day 7 postoperatively and in one study on Day 3 postoperatively. Out of the nine studies in which patients received only one injection, significant results in favour of adjuvant therapies were observed in three of them over the FU period for the subjective IKDC scale. In all three studies, the injection therapy was PRP. Statistically favourable outcomes were also noted in the VAS scale in two studies, both utilizing PRP as well. Among the three studies where patients received more than three injections, two showed significant results on the subjective IKDC scale, with PRP and MSC therapies. Considering the VAS scale, one of the studies demonstrated a significant difference in favour of the group that received PRP. There is no clear trend in clinical outcomes, based on the number of injections received or the day of the first injection.

## DISCUSSION

In recent years, orthobiologics, like PRP, MSC or HA, have gained significant interest in orthopaedic surgery [39]. These biologically derived materials aim to promote musculoskeletal tissue repair and regeneration, and while they show promise in symptom modification, evidence for true tissue regeneration remains limited [43]. Although they hold great potential, their effects are not fully understood and clinical evidence is still limited [37]. One of the main challenges in this field is the lack of standardized reporting protocols, emphasizing the need for minimum reporting standards and higher‐quality studies [39]. Despite the growing use of orthobiologics, there is a paucity of high‐quality evidence supporting their frequent application, calling for cautious optimism and further investigation [38]. PRP is the most used orthobiologic, which has proven to be a valid treatment option for knee OA and can be considered a first‐line nonoperative treatment in this context [30]. Due to its anti‐inflammatory and pain‐mitigating properties, HA injections are commonly used for knee OA, with their efficacy supported by various studies [2]. MSCs may be a promising and safe treatment for knee OA, offering pain relief and improved functional outcomes, but future research is still needed to optimize this therapy approach [54].

Almost all available studies on orthobiologics are based on research conducted in the context of OA. Therefore, the evidence regarding surgery for chondral defects is limited to date. This highlights the importance of systematically evaluating whether there are differences as compared to the results in OA treatment.

PRP was the most used orthobiologic, with the most frequent injection protocol being a single intraoperative injection. MSC were applied in four studies, while HA was utilized in only one of the included studies. In six out of the 12 included studies, a statistically significant difference was observed in at least one of the two primary measures used at 12‐ or 24‐month FU. Among these six studies, PRP was used as injection therapy in five of them, and in the other one, HA + MSC was applied. Out of the nine studies using a single injection, three reported favourable outcomes for adjuvant injection therapy compared to the control group. Additionally, all three studies employing multiple injections showed favourable outcomes on at least one scale during FU.

There is considerable variation in the type of infiltration used across studies, with most focusing on PRP. Some recent studies also report outcomes with cell‐based injections like MSC or PBPCS. Despite the findings of this review, it remains inconclusive whether any specific agent demonstrates superiority over others.

The timing of infiltrations varies significantly. In general, infiltrations are either done immediately after surgery or within 24 h, or days to weeks later. This timing difference has been considered in categorizing studies for this review. Information remains insufficient to draw clear conclusions regarding the optimal number of injections and timing of the first application for achieving favourable clinical outcomes.

Study groups receiving injection therapy in the context of cartilage repair techniques were mainly compared with groups only treated with MF. In one study, the comparison was done with groups treated differently. The study of Saw et al. [44] compared treatment outcomes between a group treated with MF + multiple injections of HA + PBSC and nonoperative treatment with multiple injections of HA + physiotherapy, reporting better results in the intervention group compared to the nonoperative group. This setup presents challenges in drawing a definitive conclusion regarding the advantage of injection therapy following cartilage repair treatment, which is why the control group was not considered for final analysis.

Safety is a crucial consideration in every treatment, highlighting the importance of reporting complications or adverse events. One study [44] documented two cases of deep vein thrombosis and some cases of progression to OA. Another study [21] reported few cases of infection, malunion of incisions and swelling and a third study [51] reported five patients experiencing pain and swelling. These findings emphasize the safety of injection therapies, which demonstrate low risk for complications.

While most studies focused on injection therapy combined with isolated BMS techniques for treating cartilage defects, a few studies involved alternative cartilage repair procedures and concomitant procedures. Danieli et al. [13] included surgeries like anterior cruciate ligament reconstruction or meniscal repair alongside cartilage defect treatment using chondroplasty with or without PRP infiltration.

To date, no studies have investigated the effect of an adjuvant infiltration therapy combined with cell‐based cartilage repair techniques such as ACI, osteochondral transplantation or minced cartilage. This gap in the literature highlights the need for research on the combined effects of injection therapy with different cartilage repair techniques.

There are some limitations that need to be considered. The studies analysed in this review exhibit important differences in various aspects, complicating direct comparisons and precise conclusions. Key factors contributing to this heterogeneity include the type of adjuvant therapy utilized, the frequency of applications and the timing of the initial injection. Additionally, variations are notable in study quality, design, sample size, FU duration, outcome measures employed, and size and grade of cartilage lesions. Furthermore, differences exist in the specific surgical techniques employed across the studies.

The way of reporting results in some studies has some limitations. Baseline data is not reported in some studies, which impacts interpretation. Additionally, in three studies, outcome measurements are presented only in figures without numerical data specification, further limiting interpretability.

Due to the limited number of studies and diversity in preparations, concentrations and products for PRP, HA, and cell‐based approaches, this review cannot differentiate between subgroups, which is a limitation.

Another important factor to consider when interpreting the results is the degree of cartilage lesion present at the time of surgery. It is noteworthy that not all studies address the same type of cartilage defects, which can vary in size or grade. Despite excluding studies where patients with advanced OA were present, there remains a wide variety of chondral defects. Most studies utilize scoring systems such as the International Cartilage Repair Society (ICRS) and Outerbridge classifications to determine the grade of cartilage lesions. Additionally, some studies report the size of the defect measured using magnetic resonance imaging (MRI). This is acknowledged as a limitation of the present review due to the heterogeneity of the lesions, which may impact the generalizability and comparability of the findings across studies.

The current evidence from the literature already demonstrates benefits of using injection therapy after cartilage lesion treatment [6, 15, 25, 45, 56, 58], but it is insufficient to determine which infiltration protocol yields the best results. Further studies are warranted to investigate the optimal orthobiologic agents, their specific preparations and injection protocols (including the number and timing of injections) that result in the most favourable clinical outcomes.

Although this review focuses on knee cartilage lesions, the ankle joint is also a common site of chondral injury and represents a relevant area for future research on ortobiologic treatments. Existing evidence suggests that although therapeutic strategies are often extrapolated from knee protocols, ankle cartilage differs structurally and biomechanically, which may influence treatment outcomes [7, 11, 29]. HA has shown effectiveness in knee OA but seems less consistent for the ankle [40]. Most ankle chondral lesions are posttraumatic, and their progression to OA appears to be less frequent than in the knee [4]. There are studies exploring the use of adjuvant injection therapies in the ankle, reporting heterogeneous protocols and outcomes, using PRP, HA or MSC [16, 18, 20, 22, 23, 27, 32, 46]. These findings highlight the need for joint‐specific approaches and further investigation into the efficacy of orthobiologic treatments beyond the knee joint.

The current evidence is limited to studies utilizing MF as cartilage repair treatment, making it difficult to draw any conclusions regarding more novel cartilage regenerative techniques. There is an opportunity for future research to explore the effects of these adjuvant therapies in conjunction with more novel cartilage treatment approaches, such as cell‐based techniques, osteochondral transplantation or microfragmented cartilage. These need to be separately investigated in RCTs to determine if it makes a difference to existing data. Such studies would contribute valuable insights into the efficacy and potential synergistic effects of combining these therapies for enhanced cartilage repair and regeneration.

## CONCLUSION

The available evidence on adjuvant injection therapies using PRP and HA after surgical cartilage repair of the knee is mostly limited to MF cases and demonstrates variable clinical outcomes, making it impossible to provide a definitive recommendation for one approach over another due to high heterogeneity. Data regarding MSC remains limited, and variations in cell harvesting and preparation protocols are noteworthy. Therefore, definitive recommendations cannot be made based on the findings from this review. Furthermore, since most studies have focused on MF as the surgical technique, no clear guidance can be given regarding adjuvant injection therapy following other, more modern procedures such as mBMS or ACT. While multiple injections indicate potential for improved outcomes, the effectiveness of specific orthobiologic agents remains uncertain due to study variability and limited comparative research. Considering existing findings from other studies, HA or PRP may be considered for symptomatic treatment in the postoperative period. HA for patients experiencing joint stiffness and poor load‐bearing capacity, and PRP for those with prolonged postoperative effusion. Nevertheless, no conclusive statement can be made regarding the qualitative or quantitative improvement of clinical outcomes.

## AUTHOR CONTRIBUTIONS

Philip P. Roessler, Philipp Niemeyer and Peter Angele conducted the initial literature search strategy and drafted the initial manuscript. Max Alfredo Saráchaga Mendoza and Alexander Bumberger reviewed full texts for eligibility and performed the data extraction. Methodological assessment was carried out by Max Alfredo Saráchaga Mendoza and Alexander Bumberger. All authors contributed to the critical reading and revision of the manuscript and approved the final version.

## CONFLICT OF INTEREST STATEMENT

The authors declare no conflicts of interest.

## ETHICS STATEMENT

In accordance with local regulations, ethical approval was not required for this study as it did not involve the use of any patient information or experimental data. Informed consent was not required for the present study, as it did not involve the use of any patient information.

## Data Availability

Data sharing is not applicable to this article as no data sets were generated or analysed during the current study.
